# Subfoveal choroidal thickness increases after excimer laser-assisted penetrating keratoplasty but not after excimer laser-assisted deep anterior lamellar keratoplasty

**DOI:** 10.1038/s41598-024-66225-4

**Published:** 2024-07-03

**Authors:** Wissam Aljundi, Loay Daas, Cristian Munteanu, Berthold Seitz, Alaa Din Abdin

**Affiliations:** Department of Ophthalmology, Saarland University Medical Center UKS, Kirrberger Street 100, Building 22, 66421 Homburg/Saar, Germany

**Keywords:** Choroidal thickness, Corneal transplantation, Excimer laser, Macular thickness, Corneal diseases, Retinal diseases, Uveal diseases

## Abstract

To evaluate the impact of excimer laser-assisted deep anterior lamellar keratoplasty (Exc-DALK) and excimer laser-assisted penetrating keratoplasty (Exc-PKP) on subfoveal choroidal thickness (SFCT) in eyes with advanced keratoconus. A retrospective comparative clinical study, which compares the outcomes of 24 eyes treated with Exc-DALK (*G1*) against matched group of 43 eyes treated with Exc-PKP (*G2*) at both 2 months (*T1*) and 2 years (*T2*) postoperatively. Main outcomes included best-corrected visual acuity (BCVA), central macular thickness (CMT), and SFCT. Preoperatively, there were no significant differences between both groups regarding BCVA, CMT or SFCT (*p* > 0.05). There were no significant differences between both groups regarding BCVA at both follow-ups (*p* > 0.05). There were no significant differences between both groups regarding CMT at both follow-ups (*p* > 0.05). SFCT was higher in G2 than G1 at both follow-ups (*p* < 0.01). Compared to preoperative SFCT, there were no significant changes in SFCT in G1 at both follow-ups (*p* > 0.05). In G2, SFCT increased significantly at T1 (*p* < 0.01) and did not differ significantly at T2 (*p* = 0.17). SFCT increased significantly after Exc-PKP but not after Exc-DALK, which might indicate that Exc-DALK affects the choroid less and thus could represent a less traumatic approach to ocular tissue than Exc-PKP.

## Introduction

Keratoconus (KC) is the most common form of primary corneal thinning and is usually defined as bilateral, asymmetric, progressive corneal ectasia leading to irregular astigmatism and visual impairment^1,2^. Depending on disease progression, a variety of treatment options might be considered, including contact lens fitting, surgical treatment with corneal cross-linking, corneal ring segment implantation, or corneal transplantation. Further progression of the disease results in severe visual impairment that cannot be corrected with contact lenses due to severe stromal thinning, scarring, and, in the worst case, acute corneal hydrops^3,4^.

Besides traditional penetrating keratoplasty (PKP), deep anterior lamellar keratoplasty (DALK) represents nowadays an alternative surgical treatment option for patients with advanced KC. The main advantage of DALK is that the host corneal endothelium is not subject to immune rejection postoperatively. Furthermore, DALK represents a closed-system procedure compared to the *open-globe technique* used in PKP and topical corticosteroids can usually be discontinued earlier postoperatively^1,5^.

In our department, patients with advanced KC are routinely treated with excimer laser-assisted PKP (Exc-PKP) or excimer laser-assisted DALK (Exc-DALK). The concept of Exc-DALK has been developed at the Department of Ophthalmology of Saarland University Medical Center and consists of an excimer laser-assisted trephination of 80% of the midperipheral corneal thickness measured with anterior segment optical coherence tomography (AS-OCT). The indications and contraindications of Exc-DALK are similar to mechanical DALK^2^.

Increased choroidal thickness might represent a *risk factor* or increase the risk of other ocular diseases such as vascular occlusion or pachychoroid disease spectrum (PDS)^6–10^. And since the choroid is an extremely sensitive tissue for changes in ocular perfusion, a postoperative increase in choroidal thickness could indicate the incidence of intraoperative perfusion fluctuations or the amount of surgical trauma produced to the ocular tissue intraoperatively^9^. Recent papers concluded, that the choroidal thickness in patients with KC is significantly higher than in healthy controls of the same age groups. This could be due to the currently widely discussed inflammatory component involved in the KC^11–14^. Since corneal transplantation represents the ultima ratio for patients with KC and the impact of different types of corneal transplantation on the posterior pole of the eye has so far only been focused on changes in macular thickness and the development of postoperative macular edema^15^, our main aim was to broaden the current evidence by investigating the behaviour of the “already increased” choroidal thickness in patients with KC after corneal transplantation. In this study, we evaluate and compare the short- and long-term impact of both Exc-DALK and Exc-PKP on subfoveal choroidal thickness (SFCT) in patients with advanced KC.

## Materials and methods

A retrospective monocentric study approved by the Ethics Committee of Saarland/Germany (*Ha 245/20*) and performed in accordance with the declaration of Helsinki. We analysed the data of all patients who underwent Exc-DALK at the Department of Ophthalmology, Saarland University Medical Center, Homburg/Saar, Germany, between January 2014 and December 2020 and included 30 eyes of 26 patients matching our below mentioned inclusion and exclusion criteria (*G1*). An Exc-PKP comparison group (*G2*) was created randomly by our statistical team after performing a pseudorandom stratified bootstrapping of the main database for corneal transplantation in our department, matching the main characteristics (KC as indication for keratoplasty, KC stage, age, preoperative tomographic properties, graft diameter and surgeon) and regarding our inclusion and exclusion criteria.

### The inclusion criteria

I. Patients of both genders aged 18 years or above, who underwent Exc-DALK or Exc-PKP at our department with a minimum follow-up of 18 months.
II. KC as indication for corneal transplantation.
III. KC stage of 3 or 4. This was classified according to Pentacam’s topographic keratoconus classification (TKC), which consists of five stages: 0 (normal), 1 (suspicious), 2 (mild), 3 (moderate) or 4 (severe KC)^16^.
IV. Central primary corneal transplantation wit Graft diameter 8.1 mm and trephine diameter of host cornea 8.0 mm.

### The exclusion criteria

I. History of any form of herpetic keratitis or keratouveitis.
II. History of acute corneal hydrops.
III. History of corneal crosslinking.
IV. Any form of previous keratoplasty.
V. Concomitant ocular or systemic pathology affecting visual acuity, central macular thickness (CMT), or SFCT, such as a history of retinal occlusion, a history of any form of uveitis, history of infectious diseases in the posterior pole, any form of diabetic retinopathy (DR) or diabetic macular edema (DME)^9^.
VI. Any disorder of the PDS such as chorioretinopathy centralis serosa (CCS).
VII. Intraoperative Descemet´s membrane perforation during Exc-DALK.
VIII. Any type of corneal vascularization.

Surgeries in both groups were performed by one of two surgeons (LD, BS) following a standardized surgical approach. In case of macro or microperforation during Exc-DALK, the operation was converted to Exc-PKP. These patients were not included in this study.

**Figure 1:**
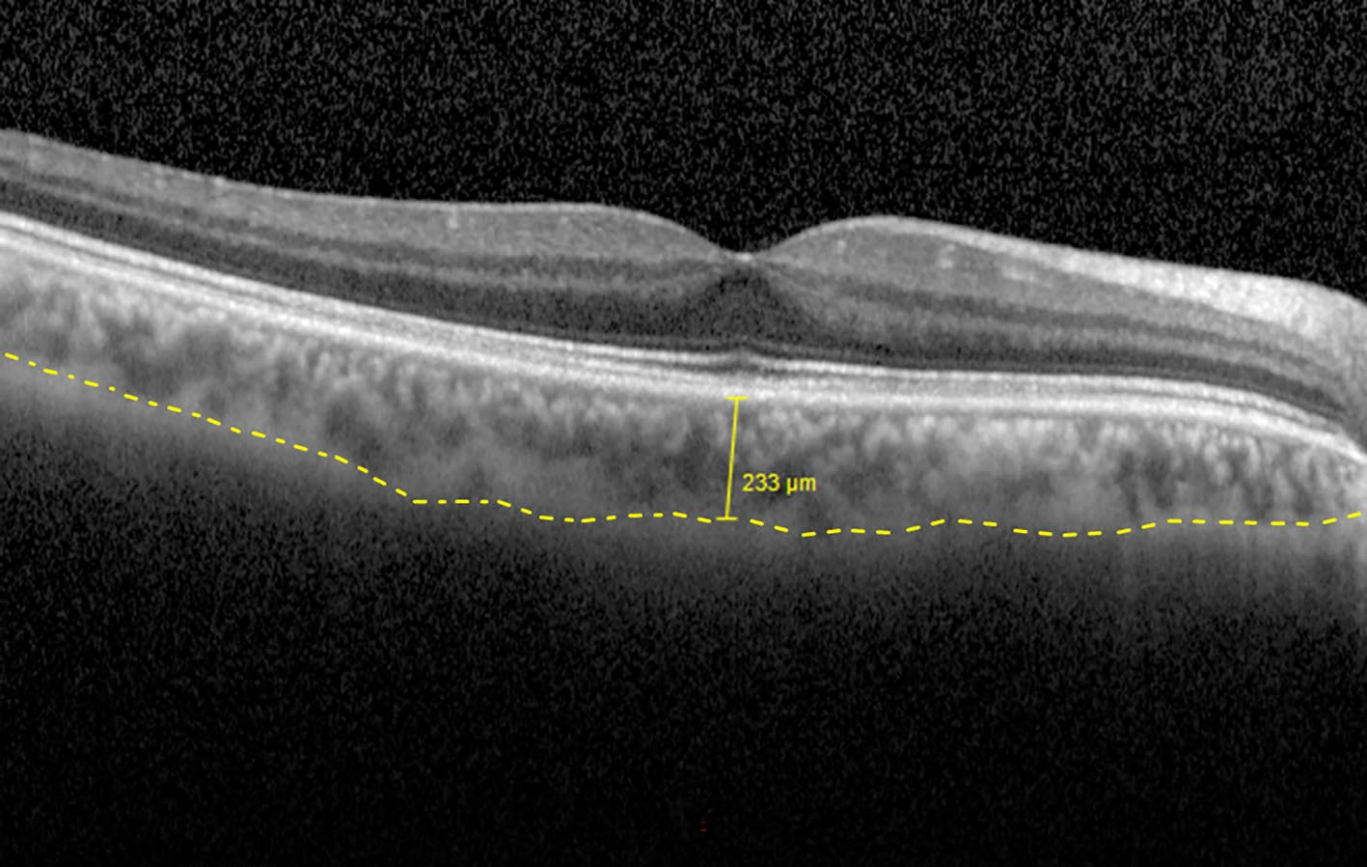
Assessment of subfoveal choroidal thickness (SFCT, µm) using enhanced depth imaging optical coherence tomography (EDI-OCT). SFCT (in µm) was defined as the vertical distance from the hyperreflective line of Bruch's membrane to the hyperreflective line of the inner surface of the sclera represented by the discontinued yellow line.

Main outcome measures included best-corrected visual acuity (BCVA, collected in decimal and converted to equivalent logMAR), CMT (µm) as measured by Spectral Domain Optical Coherence Tomography (Spectralis SD-OCT; Heidelberg Engineering, Heidelberg, Germany), as well as SFCT (µm) using enhanced depth imaging OCT (EDI-OCT; Heidelberg Engineering, Heidelberg, Germany). EDI-OCT images were taken by different technicians and analysed in a masked manner by one experienced ophthalmologist. SFCT (in µm) was defined as the vertical distance from the hyperreflective line of Bruch's membrane to the hyperreflective line of the inner surface of the sclera as shown in Fig. 1. SFCT was measured using EDI-OCT as explained, which has high intra- and interobserver reproducibility^17,18^.

### Statistical analysis

Data from medical records was collected with Microsoft Excel 2010 (Microsoft Corporation, Redmond, WA, USA) and analysed with SPSS version 26 (SPSS Inc., Chicago, IL, USA). To compare means we used the paired samples test, as well as the Mann–Whitney *U* test for non-normally distributed variables. To compare categorical variables, we used the Chi-square (χ^2^) test. To assess the behaviour of the various parameters in both postoperative follow-ups (T1 and T2), we used the Friedman test. In case of a significant difference, we compared the two follow-ups with the preoperative data using Wilcoxon test. Correlations were tested with Pearson’s correlation coefficient (r). The mean values ± standard deviation of the data are presented, and differences were considered significant if *p* < 0.05.

### Ethics statement

The studies involving human participants were reviewed and approved by Ethics Committee of the Medical Association of Saarland, Germany (*Ha 245/20*). Written informed consent for participation was waived by the ethics committee due to the retrospective nature of this study in accordance with the national legislation and the institutional requirements.

## Results

This study included 67 eyes of the Homburg Keratoconus Center (HKC) treated with Exc-DALK (*G1, 24 eyes*) or Exc-PKP (*G2, 43 eyes*). The mean age at the time of surgery (years) was 34 ± 10 in G1 and 38 ± 9 in G2 (*p* = 0.15). Based on TKC classification, KC stage was 3 in 7 eyes (29%) and 4 in 17 eyes (71%) in G1 and 3 in 15 eyes (35%) and 4 in 28 eyes (65%) in G2 (*p* = 0.22).

BCVA (logMAR) was 1.08 ± 0.38 in G1 and 0.97 ± 0.37 in G2 (*p* = 0.31). Preoperative CMT (µm) was 299 ± 49 in G1 and 302 ± 30 in G2 (*p* = 0.34). Preoperative SFCT (µm) was 320 ± 60 in G1 and 342 ± 50 in G2 (*p* = 0.06).

The first follow-up (*T1*) occurred in both study groups at about 2 months postoperatively. BCVA (logMAR) at T1 was 0.41 ± 0.23 in G1 and 0.49 ± 0.19 in G2 (*p* = 0.09). CMT (µm) at T1 was 284 ± 35 in G1 and 293 ± 27 in G2 (*p* = 0.11). SFCT (µm) at T1 was 315 ± 49 in G1 and 376 ± 42 in G2 (*p* < 0.001). The second follow-up (*T2*) occurred in both study groups at about 2 years postoperatively and after final suture removal. BCVA (logMAR) at T2 was 0.28 ± 0.09 in G1 and 0.36 ± 0.21 in G2 (*p* = 0.12). CMT (µm) at T2 was 291 ± 29 in G1 and 299 ± 21 in G2 (*p* = 0.31). SFCT (µm) at T2 was 309 ± 57 in G1 and 358 ± 43 in G2 (*p* = 0.007).

**Figure 2:**
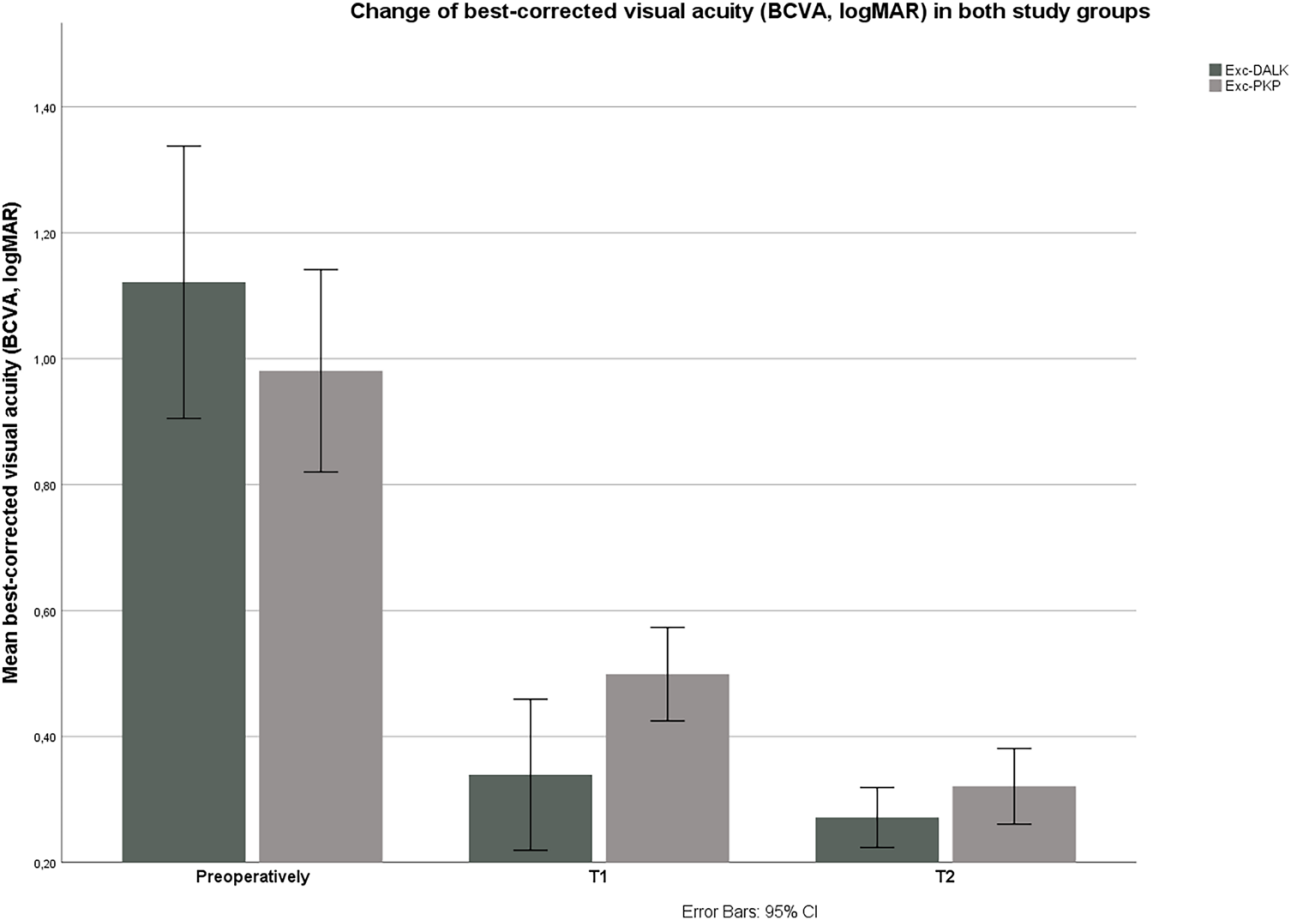
Changes of best-corrected visual acuity (BCVA, logMAR) in both groups during the observation period. There were no significant differences between the two groups regarding BCVA preoperatively, as well as at both follow-ups (*p* > 0.05). *T1* first postoperative follow-up (2 months postoperatively), *T2* second postoperative follow-up (2 years postoperatively), *Exc-DALK* excimer laser-assisted deep anterior lamellar keratoplasty, *Exc-PKP* excimer laser-assisted penetrating keratoplasty.

Postoperatively, there was a significant improvement in BCVA in both groups (*p* < 0.001 in G1 and G2, Friedman test). There was a significant improvement in both groups at both follow ups compared to the preoperative BCVA (*p* < 0.01 in G1 and G2 at both follow-ups, Wilcoxon test). The changes of BCVA in both groups are shown in Fig. 2.

**Figure 3:**
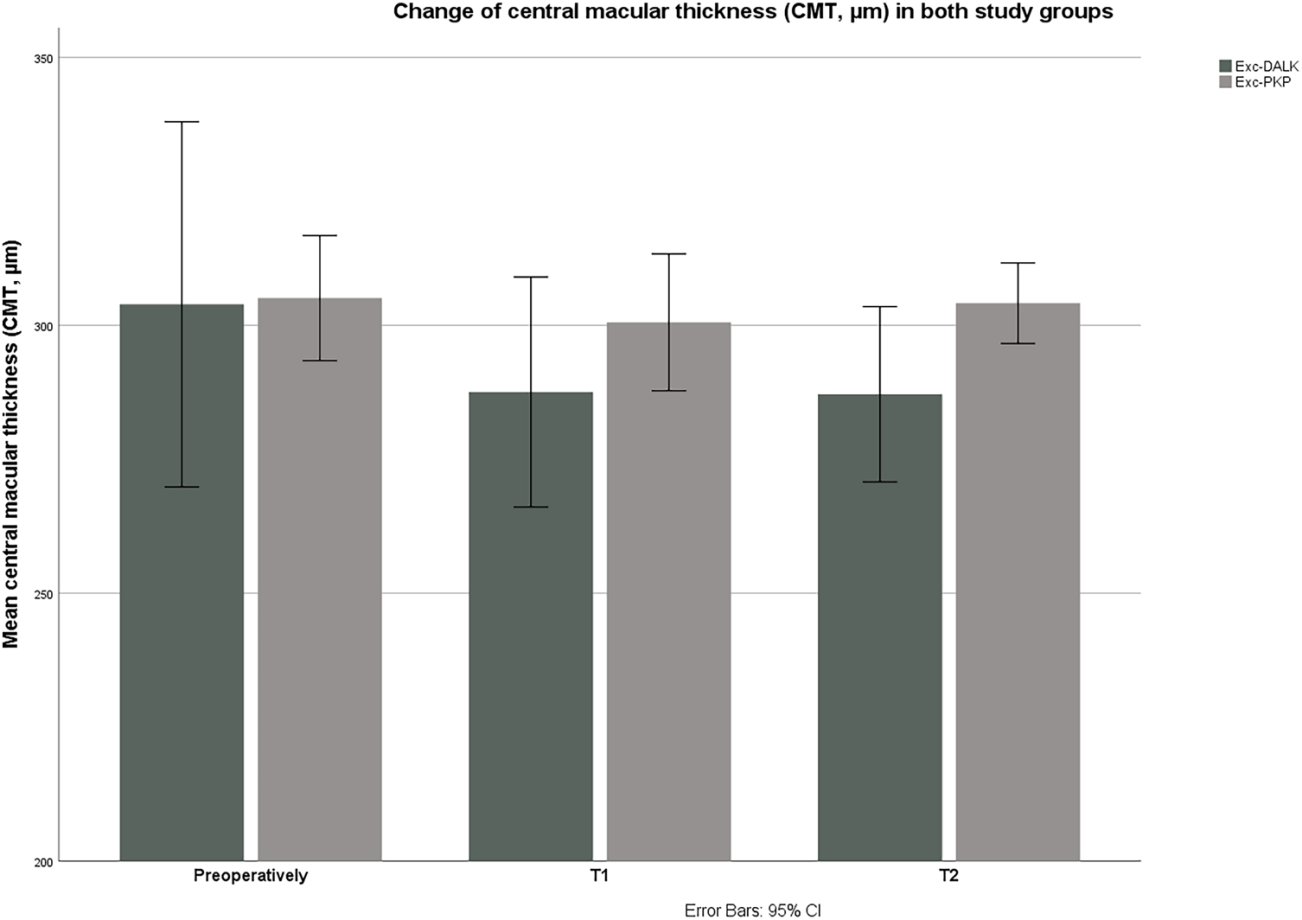
Changes of central macular thickness (CMT, µm) in both groups during the observation period. There were no significant differences between the two groups regarding CMT preoperatively, as well as at both follow-ups (*p* > 0.05). *T1* first postoperative follow-up (2 months postoperatively), *T2* second postoperative follow-up (2 years postoperatively), *Exc-DALK* excimer laser-assisted deep anterior lamellar keratoplasty, *Exc-PKP* excimer laser-assisted penetrating keratoplasty.

Postoperatively, there were no significant changes in CMT in both groups (*p* > 0.05 in G1 and G2, Friedman test). The changes of CMT in both groups are shown in Fig. 3.

**Figure 4:**
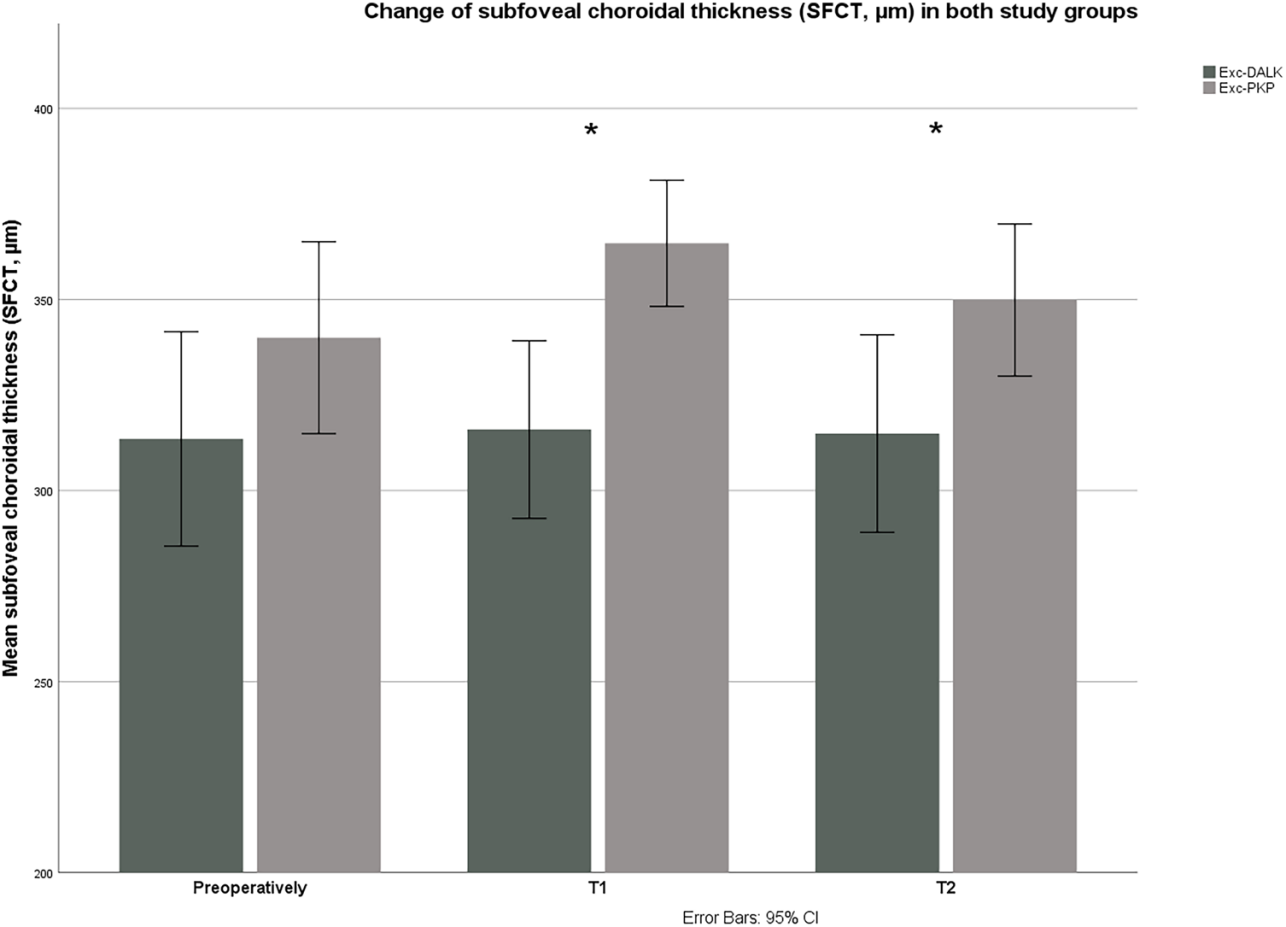
Changes of subfoveal choroidal thickness (SFCT, µm) in both groups during the observation period. Preoperatively, there was no significant difference in SFCT between the two groups (*p* = 0.06). SFCT was significantly higher in Exc-PKP group (G2) than in Exc-DALK group (G2) both at first follow-up (T1: 2 months postoperatively, *p* < 0.001), and at second follow-up (T2: 2 years postoperatively, *p* = 0.007). *Exc-DALK* excimer laser-assisted deep anterior lamellar keratoplasty, *Exc-PKP* excimer laser-assisted penetrating keratoplasty.

Regarding SFCT, there were no significant differences postoperatively in G1 (*p* = 0.62, Friedman test). In contrary, SFCT increased significantly postoperatively in G2 (*p* = 0.04, Friedman test). Compared with preoperative SFCT in G2, SFCT increased significantly at T1 (*p* = 0.01, Wilcoxon test) and did not differ significantly at T2 (*p* = 0.11, Wilcoxon test). The changes of SFCT in both groups are shown in Fig. 4.

There was no significant difference between the two groups regarding axial length (AL). AL (mm) was 24.18 ± 0.76 in G1 and 23.98 ± 1.18 in G2 (*p* = 0.48). However, there was a significant negative correlation between AL and SFCT in the overall sample (r = − 0.61, *p* < 0.001), as well as in G1 (r = − 0.53, *p* = 0.01) and G2 (r = − 0.70, *p* < 0.001) separately.

There was no significant correlation between ∆BCVA (BCVA at T2—preoperative BCVA) and ∆SFCT (SFCT at T2—preoperative SFCT) in both G1 (Pearson r = − 0.34, *p* = 0.25) and G2 (Pearson r = − 0.18, *p* = 0.95).

## Discussion

The choroid is an extremely sensitive tissue that responds strongly to ocular inflammation, trauma and changes in ocular blood flow^14^. This fact encourages us to investigate the clinically invisible impact of different types of corneal transplantation on the choroid using SFCT. This might indicate which of the two main types of corneal transplantation would produce less surgical trauma to the ocular tissues. The significant increase in SFCT in G2 about 2 months after Exc-PKP compared to preoperative SFCT represents the main finding of the present study. Contrarily, SFCT remained without significant changes in both follow-ups in G1 despite performing Exc-DALK.

Moreover, we found an increased preoperative SFCT in both study groups. This corresponds with the results of the recently published papers regarding the increased SFCT in KC eyes^11,13,14^. In 2020, Bilgin et al. reported an SFCT (µm) of 363.9 ± 59.8 in KC eyes versus 328.4 ± 67.2 in the control group of healthy eyes. Still, Pinheiro-Costa et al. found in the same year that SFCT is not a disease progression marker^14^.

The choroid is the most vascularized tissue of the eye and also the tissue with the highest blood flow per unit weight^13^. During PKP, the eye experiences a massive perfusion fluctuation especially after complete trephination, and this requires a high level of awareness on the part of the anesthesia team by actively keeping the patient in hypotension status as well as the surgeon to avoid globe decompression leading to the most devastating complication, i.e. suprachoroidal hemorrhage (SCH), which appears in 0.1% of the eyes undergoing a PKP^19,20^. To the best of our knowledge, none of the major papers on outcomes of DALK reported any SCH^21–24^. This fact alone might lead us to consider that DALK affects the uveal track and mainly the choroid less than PKP. The significantly higher SFCT observed after Exc-PKP compared to Exc-DALK in both follow-ups in short and long-term could support this assumption. Even though being very rare and not widely investigated in the literature^25–27^, this consideration could be crucial when indicating keratoplasty in a young patient with advanced KC and a history of any disorder of PDS such as CCS. For such cases, DALK might be a better option to avoid progression or decompensation of the retinal pigment epithelium (RPE) as much as possible.

Although this study included SFCT reasonably shortly after surgery at the first follow-up (approximately 2 months postoperatively), we still lack SFCT measurements immediately after surgery. This might be important as the choroid is an extremely sensitive tissue regarding changes in ocular perfusion. This lack of data is due to the technical difficulty of obtaining a good view through the still thickened corneal graft in order to properly investigate the choroid. This would be very interesting to better understand the choroidal perfusion and how the choroid behaves directly after completing a full-thickness trephination. Furthermore, it might be important to find out, whether the significant increase in SFCT we observed at the first follow-up is a slow constant increase or occurred immediately after surgery and reached its peak at some point before returning to its preoperative value at the last follow-up (approximately 2 years postoperatively). Here, further larger prospective studies, as well as improvement of the measurement requirement of EDI-OCT are necessary.

The retina as well as the blood-retinal barriers could be affected by the elevated inflammatory mediators after ocular surgery. This also affects the CMT and could lead to cystoid macular edema (CME). This aspect is well investigated after cataract surgery^28^. In contrast, the change in CMT after corneal transplantation is less examined. Genevois et al. found in 2007 that there was no significant difference in the CME ratio after PKP compared to DALK^29^. Nevertheless, the eyes studied were very heterogeneous regarding the diagnosis, the surgery performed, and the technique used. In contrast, Acar et al. found in 2011 that CMT significantly increased after PKP one week and one month postoperatively, while remaining stable after DALK^15^. According to their paper, this increase is attributed to the surgical trauma and the enhanced intraocular manipulation during PKP. Furthermore, the increase in macular thickness was attributed to an inflammatory reaction and increasing prostaglandin concentration in the vitreous cavity due to surgical manipulation after PKP. Acar et al. observed an increase in CMT after PKP reaching a peak level one week postoperatively and remaining stable until one month before returning to preoperative levels six months postoperatively. However, no patient in either group developed a postoperative CME. In our study, CMT remained with no significant change in either group in both follow-ups. Moreover, CMT was not significantly different between both groups preoperatively and in both follow-ups. This difference between the results of Acar et al. and ours could be due to the fact that we used excimer laser-assisted trephination while performing PKP, which leads to a significant reduction of surgical trauma compared to the manual trephination used in the paper of Acar et al. according to previously published papers^30,31^.

For a long time, KC was considered as non-inflammatory ectasia of the cornea, due to the lack of neovascularization or histological signs of inflammation in the excised KC corneas^32^. However, there is increasing evidence in the literature that an altered extracellular matrix and collagen types, as well as an increase in pro-inflammatory and inflammatory mediators are responsible for the development of KC^11,14,33–36^. An overexpression of inflammatory molecules, mainly Interleukins IL-1, IL-4, IL-6, IL-10, IL-17, tumor necrosis factor α (TNF-α), and Interferon γ (IFN-γ) was found in the tears of KC eyes. In addition, excessive eye rubbing, as well as wearing rigid contact lenses strongly enhances the levels of inflammation in KC eyes^32,37–40^.

These two pathomechanisms alone justify the investigation of choroidal alterations in KC. First, an altered extracellular matrix and collagen types could affect the choroid, since collagen-I, which is highly altered in KC^41^, is the main component for the media and adventitia of the vessel walls. In addition, many previously published papers concluded that the choroid appears to be very sensitive to ocular inflammation. An increased choroidal thickness was observed in the acute phase of several ocular inflammatory diseases or uveitis and, interestingly, not in the remission phase^11,42–44^. In this context, several published papers reported an increased choroidal thickness in KC eyes compared with healthy eyes^12,13,45^.

Many previously published papers concluded that the use of an excimer laser-assisted trephination leads to significantly better visual and functional outcomes after PKP compared to mechanical trephination due to better postoperative topographic astigmatism and surface regularity. Moreover, the laser-assisted trephination minimizes the exerted trauma on ocular tissue^2,30,46–48^. These advantages led us to conceptualize Exc-DALK to maintain these advantages also for the lamellar approach. In particular, laser-assisted trephination would be ideal for DALK because the trephination depth should be precisely controllable and with minimum subjective influence from the surgeon compared to mechanical trephination. Furthermore, the laser-assisted trephination would help to create “perfectly” matching incision margins and facilitate a total excision of the corneal stroma to reach the naked Descemet's membrane. This allows the patient to be able to achieve the best possible postoperative visual acuity and reduces the risk of intraoperative perforation due to the reduced manual manipulation. Moreover, these benefits are maintained, even in case of intraoperative perforation, since the excimer laser-assisted keratoplasty could be continued and the operation could be finalized as Exc-PKP given the fact that a full-thickness graft with good endothelium is available for this rescue strategy^1,2,49^.

In this study, we found no significant differences between the two study groups regarding BCVA. These results are consistent with the majority of previously published papers. A meta-analysis investigated the results of a total of sixteen previously published clinical trials involving 6625 eyes with KC that underwent corneal transplantation (1185 eyes underwent DALK and 5440 underwent PKP) and revealed that BCVA (logMAR) was not significantly different at 6, 12, 24 months follow-up between DALK and PKP^50^.

The main potential limitations of our study were the retrospective nature of the work, a relatively small population from a single medical centre, and the use of decimal visual acuity as opposed to ETDRS vision charts.

## Conclusions

Early postoperatively, mean SFCT increased significantly after Exc-PKP and did not change significantly after Exc-DALK. This might indicate that Exc-DALK as closed-system procedure affects the choroid less and thus represents a less traumatic approach to ocular tissue than Exc-PKP. However, this finding did not affect visual outcome of either surgery.

## Data Availability

The raw data supporting the conclusion of this article will be made available by the authors on reasonable request.
